# Can anaerobes be acid fast? A novel, clinically relevant acid fast anaerobe

**DOI:** 10.1099/jmmcr.0.005036

**Published:** 2016-08-30

**Authors:** Maria E. Navas, Robin Jump, David H. Canaday, Maria D. Wnek, Dhruba J. SenGupta, John R. McQuiston, Melissa Bell

**Affiliations:** ^1^​ Microbiology Laboratory, Louis Stokes Cleveland Veterans Affairs Medical Center, Cleveland, Ohio, USA; ^2^​ Geriatric Research Education and Clinical Center, Louis Stokes Cleveland VAMC, Cleveland, Ohio and Department of Infectious Diseases and HIV Medicine, Department of Medicine, Case Western Reserve University, Cleveland, Ohio, USA; ^3^​ Department of Laboratory Medicine, University of Washington Medical Center, Seattle, Washington, USA; ^4^​ Bacterial Special Pathogens Branch, Division of High Consequence Pathogens and Pathology Centers for Disease Control and Prevention, Atlanta, Georgia, USA

**Keywords:** acid fast bacilli, anaerobe, novel organism, gram variable, abscess, hospital acquired infection, Lawsonella clevelandensis

## Abstract

**Introduction:**

Anaerobic acid fast bacilli (AFB) have not been previously reported in clinical microbiology. This is the second case report of a novel anaerobic AFB causing disease in humans.

**Case presentation:**

An anaerobic AFB was isolated from an abdominal wall abscess in a 64–year-old Caucasian diabetic male, who underwent distal pancreatectomy and splenectomy for resection of a pancreatic neuroendocrine tumour. The isolated bacteria were gram-variable and acid-fast, consisting of small irregular rods. The 16S rRNA gene sequence analysis showed that the isolate is a novel organism described in the literature only once before. The organism was studied at the CDC (Centers for Disease Control and Prevention) by the same group that worked with the isolates from the previous report; their findings suggest that the strain belongs to the suborder Corynebacterineae.

**Conclusion:**

This is the fifth reported case of an anaerobic AFB involved in clinical disease; its microbiological features and 16S RNA sequence are identical to previously reported cases. Clinical disease with this organism seems to be associated with recent history of surgery and abscess formation in deep soft tissues. Acquisition from surgical material is uncertain but seems unlikely.

## Introduction

The term acid–fast bacilli or AFB is used to refer specifically to mycobacteria, which are strictly aerobic, slender, rod shaped organisms that resist decolourization with acid ethanol. Most mycobacteria are slow growing and take two to six weeks to form visible colonies on solid agar (Versalovic *et al.*, 2011). We present a case of a novel human pathogen that breaks these paradigms as it is strictly anaerobic and acid–fast.

## Case report

**Fig. 1.**
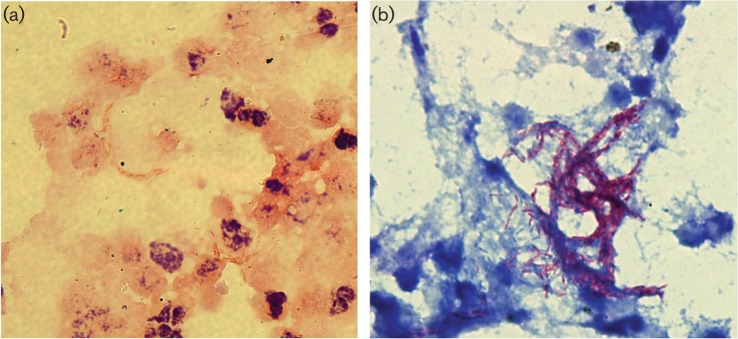
(a) Gram stain, 1000×. Direct smear of bronchoalveolar lavage showing many slender gram negative rods amidst inflammatory cells. (b) Kinyoun stain, 1000×. The bacteria stain Acid Fast Positive and show ‘cording’.

A 64–year-old Caucasian diabetic male, who underwent distal pancreatectomy and splenectomy for resection of a pancreatic neuroendocrine tumour, was seen at an outpatient clinic for follow-up one month after surgery. His surgical wound was incompletely healed and draining a fair amount of thin malodorous fluid in the absence of systemic symptoms. A computed tomography (CT) scan showed a large, complex fluid collection (21.8×9.9×24.0  cm) in the left upper abdominal quadrant (LUQ) forming a cutaneous fistula, along with a small peri-pancreatic fluid collection. The patient was hospitalized and started on ciprofloxacin and metronidazole; a superficial culture of the active drainage site grew *Staphylococcus aureus*. An endoscopic retrograde cholangiopancreatogram (ERCP) showed a large leak/fistula from the neck of the pancreas to the surgical wound and was treated with stents. The percutaneous drainage of the LUQ collection performed the same day yielded 5.3×10^9^ l^−1^ WBC. Direct microscopic examination of the fluid revealed small ‘beaded’ gram-negative rods in clumps (See Fig. 1a). In order to rule out mycobacteria, a Kinyoun acid–fast stain was performed; the organisms were fully acid–fast (See Fig. 1b) but mycobacterial cultures were negative. A second sample from the same site obtained four days later yielded the same results. Both samples were sent for universal mycobacterial and bacterial polymerase chain reaction (PCR) method at the University of Washington, but no organism could be identified. Anaerobic cultures were not performed at that time.

During the three weeks following placement of the LUQ catheter, the patient remained on broad-spectrum antibiotics showing marginal improvement but continued to have persistent drainage from the surgical wound. One month after admission, the patient became lethargic and diaphoretic with leukocytosis; the antimicrobial regimen was modified to vancomycin, meropenem, and fluconazole. No bacterial growth was obtained from the purulent drainage of his abdominal wound; however, fluid from the peritoneal drain, placed two weeks after admission, grew *Stenotrophomonas* spp. which prompted a regimen change to tigecycline.

Over the following month, the patient remained clinically stable with occasional episodes of leukocytosis and fever and an actively draining surgical wound. A CT scan performed two months after admission revealed air densities within areas of fat necrosis on the anterior abdominal wall, which prompted an exploratory laparotomy, that revealed two abscesses that were drained and cultured. Direct gram stained smears of both samples again showed fully acid–fast gram-negative rods. Anaerobic cultures yielded pinpoint, white, dry colonies after six days of incubation that routine anaerobic identification tests failed to identify. Since all efforts to identify the organism in the microbiology laboratory as well as at a local reference laboratory were unsuccessful, the isolate was sent to the University of Washington for sequencing. No matching sequence with species level identification was found in the database; however the sequence did match that of a novel organism that had been described in four different patients before (Harrington *et al.*, 2013). The isolate was then sent to the Centers for Disease Control and Prevention (CDC) for characterization and further study.

The patient remained hospitalized in the extended care facility for another five months because of the non-healing surgical wound and actively producing abdominal discharge through the drain. Antibiotic therapy with tigecycline was continued and later changed to ertapenem due to side effects; since a second peritoneal drainage sample grew *Stenotrophomonas* spp., intravenous Trimethoprim-Sulfamethoxazole was added and continued until drain removal and normalization of the WBC. During this time a stool culture looking for the anaerobic, acid–fast bacilli was performed without success. One year later, and after the patient had returned to his home with a slowly healing wound and no antimicrobials, a seroma and an incisional hernia were found and repaired during a short hospital stay. AFB stains and anaerobic cultures ordered at that time remained negative. No further complications were noted after this hospitalization. The patient has returned regularly for post-operative check-up visits with no further complications and no tumour relapse through 2015.

The patient had history of morbid obesity treated with bariatric surgery in 1987; in spite of this, his body mass index (BMI) was 36 before being admitted to the hospital. He also battled with diabetes and complications of the disease and had a hard time maintaining good glycaemic control; his glycated haemoglobin, i.e. HbA1c was IFCC 91.3 mmol m^−1^ (DCCT 10.5 %) around the time of his admission.

The patient was a male veteran living in a small rural town of Ohio who made a living as a truck driver, although he had been out of a stable job for five years. During those last five years he did odd jobs, most of them related to driving and transportation. The patient lived in a small rural home with electricity, gas services and water obtained from a pond; he shared his dwelling with two healthy cats. He led a sedentary lifestyle and was on a diet consisting mostly of ready-cook meals and simple to prepare dishes; he did not consume unpasteurized milk, raw meats or eggs. He had not travelled outside of Ohio for more than ten years. His family history and hobbies were not contributory.

## Investigations

The morphological and staining characteristics (size, Gram’s stain, and modified Kinyoun’s acid-fast), optimal growth temperature, and oxygen requirements of the strain were determined at the CDC from growth observed at 14 days. They were tested at 25, 35, and 45 °C in air, in a candle jar, with a Campy Pak (BD), and in anaerobic conditions (anaerobe jar and anaerobe chamber) on CDC anaerobic blood agar (BD). Cell shape and staining characteristics were observed using a Zeiss light microscope at 1000×.

Genomic DNA from the strain was purified using the Epicentre Metagenomic DNA Isolation Kit for Water (Illumina, Madison, WI). A 1441 bp fragment of the 16S rRNA gene was amplified and sequenced as previously described (Lasker, 2011). The 16S rRNA gene sequence was analyzed using the Basic Local Alignment Search Tool (http://blast.ncbi.nlm.nih.gov/Blast.cgi).

Colonies were pin-point and grew to 2–3 mm in diameter becoming visible after seven days. Cells were gram-positive and partially acid-fast, consisting of small irregular cocci with an occasional short rod. With age, cells became gram-variable, still partially acid-fast, and mostly bacillary up to 2 µm in length. Strains could not be maintained in broth or semisolid media. Growth was obtained under CampyPak and anaerobic conditions at 25 and 35 °C. The strain grew best in an environment with an oxygen tension at less than or equal to 1.0 % at 35 °C. The 16S rRNA gene sequence analysis showed that the strain belongs in the suborder Corynebacterineae and the most closely related phylogenetic neighbour is *Dietzia timorensis* DSM 45568T (95 %) (Lasker, 2011).

## Discussion

This is the fifth reported case of soft tissue infection and abscess formation with this peculiar organism; no clinically relevant anaerobes described in the literature have ever been reported as being AFB (Versalovic *et al.*, 2011; Mahon *et al.*, 2011). The patients described in all cases had recent abdominal surgery and, except in one case, the infection was limited or contiguous to the subcutaneous adipose tissue. Another common characteristic among the subjects was obesity and some association with cancer treatment and diabetes, both of which could predispose to immune dysfunction.

The course of the disease was protracted with a subacute presentation that suggests the infection was due to a low virulence pathogen, probably acquired from the surgical wound and forming a deep fistulous abscess. Similar to the other reported cases, the lack of wound healing was the most challenging aspect of the treatment and an important contributory factor to the clinical complications in this patient. Antimicrobial therapy appears to have played very little role in the recovery of the patient, rather, as with most abscesses, their drainage is what led to clinical improvement.

Cultures from blood and respiratory sites remained negative, drain fluid cultures yielded a different organism; however, its relationship to our pathogen was non-contributory and its presence a source of confusion, as it was likely a drain colonizer. The organism was isolated only from aspirated abscess contents, which supports the notion that this is an abscess-forming strict anaerobe. This case illustrates the fact that fluid obtained from drains (peritoneal fluid in this case) is of little value for microbiological studies (Everts *et al.*, 2001). The question of whether this organism is part of the normal skin flora is, however, still unanswered.

There is a relationship to abdominal surgery in most of the cases which raise the possibility that the organism is part of the normal gut flora. A stool culture looking for this organism was attempted without success; this however, does not eliminate the possibility that the organism is part of the normal gastrointestinal microbiota. The organism is difficult to grow and might need special growth conditions that were not available for our screening of the stool.

A history of sick contacts or risk factors for infection related to social history was not evident. There is not much reported about the dwelling characteristics of the other cases, and in this case they do not appear to be clearly significant; water supply from a pond is slightly unusual but its relevance unknown.

No peculiarity or unusual event was found in the review of the pancreatic surgery of this patient nor was it reported in any of the previous cases; the possibility of an organism acquired from hospital equipment or environment seems to be low.

In the clinical microbiology laboratory the organism behaved in a fashion very similar to that of the previously described cases. Our isolate along with the isolates previously described in the literature were studied at length at the CDC, confirming that they represented the same organism. Interestingly, our isolate was fully acid–fast in our laboratory, but when studied at the CDC along with the other isolates, it was categorized as partial acid–fast; this might be explained by differences in staining technique and reagents amongst laboratories. The direct gram stained smear of the sample was strongly suggestive of the presence of an infectious organism as evidenced by the presence of many white blood cells and bacteria with a single morphology (See Fig. 1a). Primary culture and subculture in the clinical laboratory were challenging; the minimal incubation time was six days at 35 °C employing a pre-reduced Brucella Agar (Oxyrase) and an anaerobic growth system (GasPak; Becton Dickinson). The morphology of the colonies grown in the clinical laboratory resembled that of the morphology described by the CDC. None of the automated methods and chemical panels used for anaerobic identification in the clinical laboratory yielded results that could suggest biochemical similarities to any of the known anaerobic pathogens. Interestingly, the closest phylogenic relative of this organism, *Dietzia timorensis*, is a recently described organism found in soil but not described as anaerobic, acid–fast or associated with human disease (Yamamura *et al.*, 2010); however, other *Dietzia* spp. have been reported as potential nosocomial pathogens associated with foreign material or disease agents in the immunocompromised (Pidoux *et al.*, 2001; Gharibzahedi *et al.*, 2014).

In conclusion, this novel organism is an abscess forming anaerobe that causes a localized infection associated with previous history of surgery. The number of reported cases is too small to determine potential risk factors; however, obesity stands out as an important characteristic. Although a previous history of malignancy or immune dysfunction was reported in all the cases, there were, however, no specific details (type of malignancy, treatment, etc.) that might definitively link them all. The available data on the reported cases does not allow us at present to formulate hypotheses on whether the organism is acquired from the environment or part of the normal human flora.
